# EEG Differentiation Analysis and Stimulus Set Meaningfulness

**DOI:** 10.3389/fpsyg.2017.01748

**Published:** 2017-10-06

**Authors:** Armand Mensen, William Marshall, Giulio Tononi

**Affiliations:** ^1^Center for Sleep and Consciousness, University of Wisconsin-Madison, Madison, WI, United States; ^2^Department of Neurology, Inselspital Bern, Bern, Switzerland

**Keywords:** event-related potentials (ERPs), differentiation, EEG, meaningfulness, images

## Abstract

A set of images can be considered as meaningfully different for an observer if they can be distinguished phenomenally from one another. Each phenomenal difference must be supported by some neurophysiological differences. Differentiation analysis aims to quantify neurophysiological differentiation evoked by a given set of stimuli to assess its meaningfulness to the individual observer. As a proof of concept using high-density EEG, we show increased neurophysiological differentiation for a set of natural, meaningfully different images in contrast to another set of artificially generated, meaninglessly different images in nine participants. Stimulus-evoked neurophysiological differentiation (over 257 channels, 800 ms) was systematically greater for meaningful vs. meaningless stimulus categories both at the group level and for individual subjects. Spatial breakdown showed a central-posterior peak of differentiation, consistent with the visual nature of the stimulus sets. Temporal breakdown revealed an early peak of differentiation around 110 ms, prominent in the central-posterior region; and a later, longer-lasting peak at 300–500 ms that was spatially more distributed. The early peak of differentiation was not accompanied by changes in mean ERP amplitude, whereas the later peak was associated with a higher amplitude ERP for meaningful images. An ERP component similar to visual-awareness-negativity occurred during the nadir of differentiation across all image types. Control stimulus sets and further analysis indicate that changes in neurophysiological differentiation between meaningful and meaningless stimulus sets could not be accounted for by spatial properties of the stimuli or by stimulus novelty and predictability.

## Introduction

**FIGURE 1:**
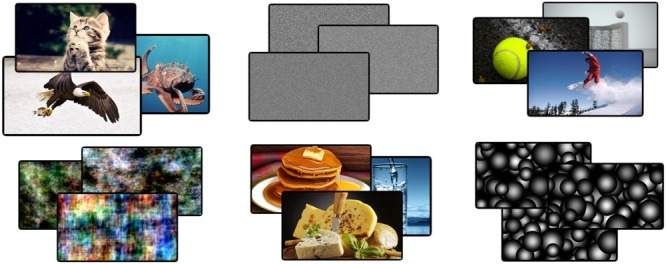
Example images from four categories. In total 34 unique images were used from 10 different categories. Examples are shown from the ‘animals,’ ‘noise,’ ‘phase-scrambled,’ ‘food,’ ‘sports,’ and ‘disks’ categories. These categories were grouped into meaningless and meaningful sets of images. Meaningful categories contain both high- and low-level features that are phenomenally distinguishable, whereas meaningless sets do not.

Consider seeing two images of particular person’s face; in one image the individual is smiling and in the other frowning. Our experience of these images will be meaningfully different although the physical properties of the images may only have changed marginally. Now consider seeing two images of unique television noise. Our experience of these images will be essentially identical despite there being no correlation between the images. This principle can be extended to whole sets of images like those in **Figure 1**. While each image set will differ in some low-level features (e.g., pixel-to-pixel correlation), only those sets with distinct higher-level invariants (e.g., cat/octopus/eagle), would we consider to be meaningful. We therefore define meaningful differences as any phenomenally distinguishable aspect of the images. Meaningfulness thus also lies on a continuum whereby the more distinguishable ideas between stimuli, the more meaningfully different they are. Assuming that phenomenal differences must be supported by some differences in the activity patterns of the brain, measures of neurophysiological differentiation could be used to index the meaningfulness of a set of stimuli for any individual subject.

Previously we assessed stimulus meaningfulness applying this principle of neurophysiological differentiation using functional magnetic resonance imaging (fMRI). Differentiation of brain responses, measured as their Lempel-Ziv complexity (compressibility), was significantly higher for meaningful movie clips (Charlie Chaplin) than for television noise, despite comparable levels of overall neural activity (Boly et al., 2015). Here we further develop an index of neurophysiological differentiation that can be applied to brain responses measured using high-density electroencephalography (EEG). Moreover, we analyze EEG differentiation for a much larger set of stimuli and control conditions.

As a proof of concept for EEG differentiation analysis (DA), we contrasted two trivial broad sets of images: natural, meaningful images and artificial, and meaningless images. Meaningful stimuli were taken from seven categories of natural images: animals, buildings, cars, food, household objects, people, and sports. Meaningless stimuli were constructed artificially within three categories: television noise, phase-scrambled natural images, and overlapping random disks, the latter two to control for the lack, in television noise, of second-order spatial properties (e.g., lines and edges) to which EEG activity may be particularly sensitive (Scholte et al., 2009; Groen et al., 2013). As the example in **Figure 1** illustrates, however, phase-scrambled images are nonetheless appreciably meaningfully different with respect to their low-level properties. Given the inherent variability and low signal-to-noise ratio of EEG signals at the single-trial level, responses to sets of meaningless images provide an empirical baseline against which to compare the differentiation elicited by meaningful stimuli. Finally, we employed several additional controls to account for various potential confounds. Since surprise can have a significant effect on EEG activity (mismatch negativity; Fishman, 2014), we manipulated the predictability of the upcoming image by presenting different cue types prior to the target image presentation. Finally, we manipulated the short-term novelty of stimuli through prior habituation of a subset of images and the long-term familiarity of the image through the collection of behavioral ratings (Barto et al., 2013). Given the clear distinction between meaningful and meaningless images used, we expect the differentiation measure to be relatively higher in the former at both the individual and group levels with similar spatio-temporal patterns.

## Materials and Methods

### Participants

In total, 9 male participants between 24 and 29 years of age completed the experiment. All participants had normal or correct vision and reported no neurophysiological or psychiatric history. Participants were given a verbal description of the experiment, as well as an information sheet and were required to sign a written consent form approved by the Health Sciences Institutional Review Board of the University of Wisconsin–Madison.

### Stimuli

A total of 340 images were obtained for the experiment (34 unique images per category). These were split equally into 10 broad categories of which 7 were considered to be meaningful and 3 generally meaningless. Meaningful categories consisted of natural images of: animals, buildings, cars, food, household objects, people, and sports. The remaining three sets of images acted as meaningless control images. The primary criteria for appropriate control images is minimal phenomenal differentiability. To this end, images of random pixel noise are a clear baseline as any two images are virtually indistinguishable after brief presentations, especially with any considerable temporal gap between presentations. Random pixel noise was created using Matlab’s ‘rand’ function over the three color layers of the image, producing noise images within a range of colors as opposed to classic gray-scale noise. This set of random noise images is, by design, absent of higher-order image statistics and any neurophysiological differences may be attributed to this basic distinction. Two additional meaningless image sets were created with minimal phenomenal differentiation, but which nonetheless displayed some range of higher-order image statistics. ‘Dead leaves’ images consisted of medium sized, opaque, three-dimensional spheres overlaid on each other (Bordenave et al., 2006). This particular configuration was chosen because they have high-order spatial statistics in the same range as the natural images selected (Groen et al., 2012). For the third meaningless image set we took a random subset of the natural images and phase-scrambled them. This was done by separating the phase and magnitude of the images, adding a random phase between 0 and 2 pi, randomly rotating magnitude quadrants and mirroring, then recombining magnitude and phase to form the new image (Honey et al., 2008). The resulting images had a low contrast energy with blurred edges while maintaining some clear spatial, higher-order image properties which contrasted well with the high contrast energy dead leave image set. While other forms of image distortion have been developed which render the particular objects in an image unrecognizable, they tend to still be readily phenomenally distinguishable from one another (Stojanoski and Cusack, 2014). It is always possible that some additional image statistic, not explicitly explored here, could distinguish meaningful and meaningless images and these should be the topic of future investigation. However, care must be take due to the likely confound between the order of image statistics present and phenomenal distinguishability.

Predictability was controlled by displaying one of three visual cues prior to the actual target image. This cue could either be a “?” and thus uninformative, the name of the category from which the image came, e.g., “car,” or a filtered version of the target image showing only the detected edges. Edge detection was performed using ImageMagick (tm), using a radius filter from 0.5 to 1. The categorical cue (category name), should in theory allow for the participant to generate a semantic template of the target image, and thus recognition may be improved. The edge-image cue allows for the near complete prediction of the upcoming image, often including its high-level category.

Short-term novelty was controlled by repeatedly showing a subset of 20 images from the meaningful categories (chosen randomly for each participant). Each habituated image was shown to the participant a minimum of 15 times while preparing the electrode net. The number of habituated images was set at 20 as a balance between having sufficient images, and thus event-related potentials, for reliable comparisons while also ensuring that the number of images was not too many to prevent perfect recognition. Finally, long-term novelty was measured by asking the participants to rate the familiarity of the target image on a scale of 1 to 3. In order to induce a range of familiarity values the selection of the experimental images attempted to find both common and rare category representatives; e.g., a dog versus a camel, a pick-up truck versus a concept car.

### Task

**FIGURE 2:**
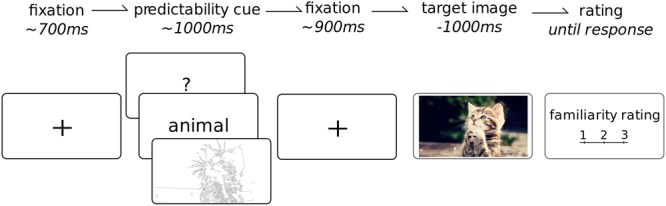
Task overview. Each trial began with a fixation cross (duration jittered in time), followed by a cue which provided potential information about the upcoming image. This was followed by another fixation point (jittered duration). The target image, from 1 of 10 categories, was then shown for 1 s. On 25% of trials, the participant was required to rate the overall familiarity of the preceding target image from 1 to 3. Each participant completed a total of 400 trials.

See **Figure 2** for an overview of the task. Each trial began with a black fixation cross over a white background for 700 ms (jittered to 600–800 ms); followed by 1 of the 3 cue types for 1 s; then the fixation cross again for another 1 s (jittered to 900–1100 ms); the target image was then presented for 1 s. On a quarter of the trials (randomly selected), after the presentation the participant was asked to rate the familiarity of the preceding image. This was only done on a quarter of the images for two reasons. Firstly we wanted to minimize the impact of this task on the attention given to each image such that the presentation of the target image remained fairly passive. Furthermore, given this was not the main aim of the study, reducing the number of ratings allowed for the presentation of a larger number of total images. The habituated images were repeated four times and the novel images only once, leading to a total of 400 images which, on average, required the attention of the participant for about 40 min. The cues and images were presented in a fully randomized sequence. The cue images were balanced across the experiment and equally likely to occur for any particular category. The task sequence was programmed and images were presented using Psychtoolbox (Brainard, 1997), within the Matlab (version 2012a) environment to ensure precise timing of the stimulus onset. Participants were seated comfortably at a viewing distance of 60–70 cm in front of a 19′ monitor. Each picture had a set resolution of 1280 by 720 pixels which fully occupied the horizontal space of the screen. Participants were instructed to maintain fixation at the center of the screen and to try to refrain from blinking during the target image. Following each familiarity rating, the participants were encouraged to take a quick break from the task, even just for a few seconds, to regain focus and attention as well as ask questions or comment on the experiment. A longer break was enforced after 200 pictures were presented.

At the end of the experiment, participants were asked to rate how distinct the individual images within a category were. This was done for each category based on their low-level and high-level features. Examples were given to indicate the kind of features that defined low and high-level aspects of the images such as hair color/head shape for low-level and age/gender for high-level. This rating was taken as an estimate of the behavioral differentiation of each category to compare to neurophysiological differentiation measures from the EEG.

### EEG Recording and Processing

Electroencephalography activity was recorded using a high-density, 256 channel (with central reference and posterior ground channel), electrode net from EGI Geodesics (Tm), and the NetAmp-300 amplifier sampled at 1000 Hz. Impedance was kept below 50 ohms for all electrodes. Triggers from the experimental computer were sent directly to the amplifier using the parallel port and EGI supplied cable to ensure a constant low latency in event trigger placement in the data. Preprocessing of the time series data was done using a combination of EEGLAB (Delorme and Makeig, 2004) and custom scripts in Matlab. Each recording was first down-sampled to 250 Hz, then bandpass filtered from 0.5 to 40 Hz. Data was then segmented around the target images, initially from 500 ms prior to image onset to 2 s after. The data was then manually inspected for bad channels (mean number of removed channels = 5.2, *SE* = 1.3) and bad epochs which were then subsequently removed from that data (mean number of trials = 365.8, *SE* = 11). Independent components were then manually examined using their topography, temporal evolution for each trial, averaged ERP, and frequency spectra for components. Components relating to eye blinks, eye movements, muscle artifacts, and heart beats as well as any other technical artifact were removed (mean components removed = 88.1, *SE* = 4.8). Once these components were removed the epochs were further cropped to 200 ms prior to image onset to 1000 ms after, and again manually inspected for any remaining bad epochs. Finally, any channels that had been removed were re-introduced using topographic spline interpolation, and the data was re-referenced to the average activity of all channels; and the original reference channel (Cz) re-introduced to the dataset.

Statistical comparisons between the ERP waveforms of the various factors were performed using a mass-univariate approach of repeated measures analysis of variance (ANOVA) or for single factor comparisons paired *t*-tests conducted independently for each channel and sample between conditions. The threshold-free cluster-enhancement (TFCE) procedure combined with the maximum permutation technique was then used to determine whether these values were significantly different for conditions across the participant while controlling for multiple comparisons. This approach examines the individual values based on their surrounding neighborhood, in both time and space, such that those values which are well supported by their neighbors are enhanced and those that are not are suppressed (Smith and Nichols, 2009; Mensen and Khatami, 2013). To test for statistical significance, the same procedure was applied to randomly permuted datasets of the original datasets 5000 times. For each permuted dataset the maximum statistically enhanced value was taken from the entire dataset, regardless of channel or sample, to form an empirical distribution of maximum permutation statistics from which to compare the original dataset. A *p*-value is obtained for each channel-sample pair by determining the proportion of the empirically derived statistics which is greater than the value for that particular channel-sample pair. This procedure has been shown to be statistically valid, robust to various signal shapes, and more powerful than traditional methods (Mensen and Khatami, 2013; Pernet et al., 2015).

### Differentiation Analysis

**FIGURE 3:**
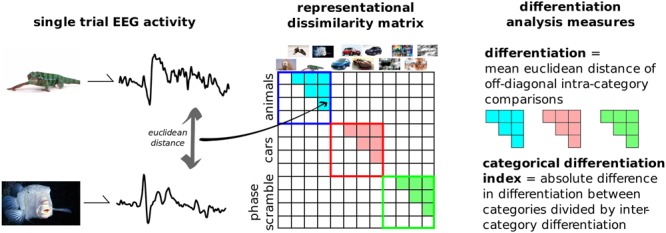
Differentiation analysis overview. The evoked responses from each presented image is compared to every other. Distances were computed using the Euclidean distance in the multi-dimensional space of channels and samples. The resulting representational dissimilarity matrix can then be split into separate categories; here into the 10 predefined image groups shown in **Figure 2**. Differentiation to each category is the mean Euclidean distance across trials from within that category. The categorical differentiation index (CDI) is a summary measure between any two categories which is normalized by between-category distances. For example, here CDI is the comparison between meaningful versus meaningless image sets.

**Figure 3** outlines the principle steps involved in DA. This novel analytical technique, introduced here, aims to quantify the differences in neurophysiological activity evoked by a set of stimuli. It is a multivariate analysis, where the relationships between several dependent variables are captured but left unspecified (Sandberg et al., 2014). EEG activity across channels and time, time-locked to the onset of a stimulus is considered as a single *state* of the brain. The differences between each of these states are then compared using some distance metric in this high-dimensional space. These distances can be placed in an n-by-n matrix generally referred to as a representational dissimilarity matrix (RDM). DA defines neurophysiological differentiation to a set of stimuli as the mean distance in the RDM between stimuli *within* that set. In this way it differs from representation similarity analysis (RSA), as this primarily focuses on differences between categories and the comparisons of multiple RDMs from distinct sources, such as neurophysiological activity, behavioral, computational models, etc. (Kriegeskorte et al., 2008; Kriegeskorte and Kievit, 2013).

Distance measures can be broken down into two broad categories: measures that emphasize the absolute distances in these high-dimensional state-spaces, such as Euclidean distance, and measures that are sensitive to the pattern of the signals such as correlational distance. As with previous EEG studies using RDMs, we use the Euclidean distance to compare EEG state-spaces (Groen et al., 2012, 2013; Walther et al., 2015). This is because EEG signals are primarily driven by the synchronized activity of large groups of neurons within local patches of the cortex, the general pattern of the ERP is likely to be very similar across the stimuli, whereas the differences between images will likely be subtler amplitude and/or topographical changes riding on top of this common ERP (see Supplementary Figure 1 for comparison of Euclidean vs. correlational distance measures). The point-to-point comparison between states in DA increases specificity compared to variance, while requiring fewer samples to estimate than entropy (Shannon, 1948); the additive nature of even small distances in the multivariate space increase its sensitivity compared to the binarization required for Lempel-Ziv complexity measures (Casali et al., 2013).

We applied DA to this dataset by considering all 800 post-stimulus time points over the 257 recorded channels as the single states to be compared, and measuring the differences using classic Euclidean distance. We then split the RDM into 10 categories based on image content without presupposing which types of images were meaningful to the participants. Furthermore, a single, summary measure was calculated by subtracting the differentiation values to artificially generated, meaningless images (disks, noise, and phase-scrambled), from the real-world, meaningful categories (animals, buildings, cars, food, objects, people, and sports). This value was normalized by dividing it by the mean between-category differentiation values. Positive values of this measure, dubbed the category differentiation index (CDI), indicate higher differentiation to meaningful stimuli sets.

Significance at the individual level was calculated using a permutation approach applied to the dissimilarity matrix. The CDI was calculated for the original dissimilarity matrix, then the image labels were randomly shuffled to create a new dissimilarity matrix and the CDI was calculated again. This re-sampling process was repeated 5000 times to obtain an empirical distribution of CDI values against which to compare the observed value. The *p*-value is equal to the proportion of values in the empirical distribution that are more extreme than the observed value.

## Results

### Neurophysiological Differentiation and Spatio-Temporal Breakdown

**FIGURE 4:**
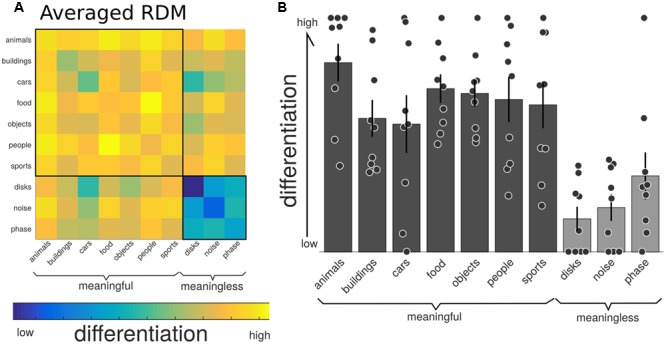
Neurophysiological differentiation to image categories. **(A)** The representational dissimilarity matrix (RDM) showing the mean distances across the trials within that category, averaged over all participants (*n* = 9). The diagonal of this averaged matrix is the differentiation value given to each category. Note the last three phenomenally meaningless categories show the lowest overall differentiation. **(B)** Mean differentiation to each category across all participants (equal to the diagonal of the averaged RDM in **(A)**; with error bars indicating the standard error and individual markers for each participants differentiation level for each of the stimulus sets. Phenomenally meaningful image sets show significantly higher differentiation compared to meaningless image sets at the group and individual levels. Moreover, the specific pattern of differentiation across the 10 categories correlated with the participants behavioral ratings of perceived similarity.

**Figure 4** shows the group-average distance matrix across all categories. The significance of group level effects was assessed using non-parametric permutation statistics (1000 permutations), on the linear mixed model with meaningfulness used as the predictor to individual categorical differentiation. The meaningful categories had a higher, normalized, differentiation value compared to the meaningless categories (mean difference = 0.436, *SE* = 0.05; *T* = 7.56, *p* = 0.001). The same test using the individual category label revealed a significant omnibus effect (*F* = 8.243, *p* = 0.001). *Post hoc* comparisons were performed using a permutation analysis of the mean difference between each pair of categories. Here, the number of unique permutations possible is 512 (2ˆ9), thus limiting the *p*-value to 0.002. Since this lower bound is already above the alpha level needed to determine significance after correction for the 45 unique comparisons (0.05/45 = 0.011), we are unable to make definitive statements about which specific categories were significantly different from which other. The results of these *post hoc* permutation tests are shown in Supplementary Figure 1.

**FIGURE 5:**
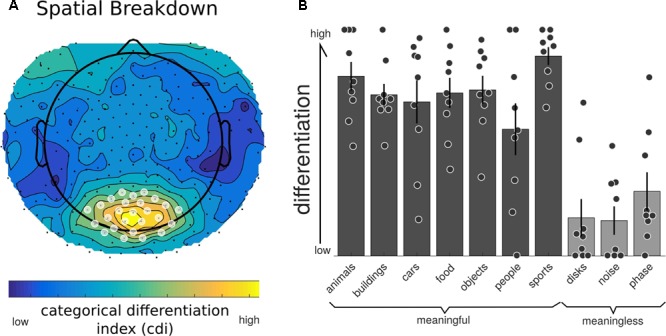
Neurophysiological differentiation for individual electrodes. To investigate whether any electrodes contributed to differentiation in particular, the averaged RDM was calculated at each individual electrode (using the Euclidean distance across trials for all time points). **(A)** All channels showed a positive CDI; indicating higher differentiation to phenomenally meaningful image sets compared to meaningless ones. Electrodes in the central-posterior region showed particularly high values and were chosen as a region-of-interest for further analyses (marked with a white overlay). **(B)** Individual differentiation values for the posterior region of interest indicated in **(A)**. All phenomenally meaningful image categories showed higher differentiation values than meaningless images sets.

We then examined both the spatial and temporal breakdown of differentiation to determine whether there were particular channels or time ranges that contributed most markedly to differentiation. For the spatial breakdown (see **Figure 5**), individual RDMs were constructed for each channel over the entire time course of the stimulus presentation and CDI was calculated as before. Significance was measured using the non-parametric TFCE procedure. Two clusters of channels had significantly positive CDI values, indicating higher neurophysiological differentiation to the meaningful stimulus sets. The largest channel cluster included 65 channels spread over the central-posterior region of the head. The peak of this region was found at channel E124 (central-posterior; mean CDI = 0.103, *t* = 9.545, *p* < 0.001). A smaller, frontal set of 36 channels also had significantly higher differentiation for the meaningful stimuli (peak channel: E224; mean CDI = 0.034, *t* = 8.836, *p* = 0.007). A region of interest was created using a subset of the most significant channels (*p* < 0.005). This ROI consisted of 20 channels from the central-posterior region.

**FIGURE 6:**
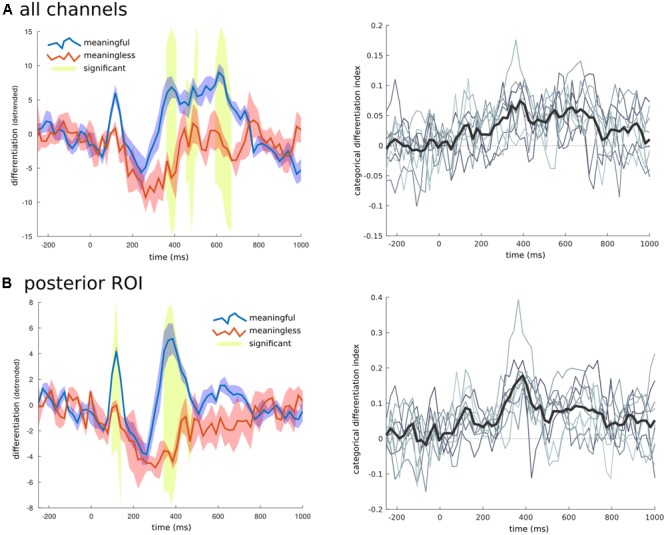
Temporal breakdown. To investigate whether certain time ranges contributed to the overall differentiation effects found, the averaged RDM was calculated for consecutive windows of 20 ms. **(A)** When all electrodes were examined three later time ranges were found to have significantly higher differentiation to phenomenally meaningful images compared to meaningless ones. **(B)** When only the channels for the posterior region of interest (shown in **Figure 5**), were used to create the RDM, an early time range around 110 ms, and another later period between 350 and 480 ms were found to have significantly different levels of differentiation between the meaningful and meaningless image categories. On the right side is the time series of the CDI (difference between the within-category differentiation of meaningful and meaningless image categories normalized by the between differences), at both the group (thick black line), and individual levels (thin gray lines).

To examine the time domain, a unique RDM was created for consecutive short time windows of 20 ms spanning both the baseline and full second of image presentation. For each RDM the differentiation value for each category was calculated along with the CDI and significance using the same permutation approach applied to the spatial domain. When all the channels were considered together (**Figure 6**), there were three time ranges where the CDI was significantly different from zero: from 385 to 405 ms (*t* = 5.763, *p* = 0.015); from 465 to 510 ms (*t* = 5.720, *p* = 0.023); and from 630 to 670 ms (*t* = 5.006, *p* = 0.043). When examining the temporal breakdown in the posterior ROI we again found significant time points around the 385 ms mark (*t* = 8.631, *p* < 0.001), but also an earlier peak at 115 ms (*t* = 5.179, *p* = 0.039).

### Individual Differentiation and Behavior

Focusing on the central-posterior region of interest, all participants showed significantly higher differentiation for meaningful stimuli across time and also for the group temporal peak at 385 ms (*p* < 0.05). At this ROI, the earliest temporal peak (90–130 ms) showed significant individual results in 7 of 9 participants. When examining all channels and time points together, of the 9 participants, 6 showed significantly higher neurophysiological differentiation (*p* < 0.05), for meaningful images while 2 showed trend levels (*p* < 0.1). In the time domain over all channels, 7 of the 9 participants showed significant CDI at the 385 ms mark, while 5 showed significant CDI at later time points. These results are crucial as they demonstrate the feasibility of applying DA at the individual level.

To examine whether the differentiation of individual categories was related to participants’ behavioral judgments, we performed permutation analysis using the individual participants ratings as predictors. Both the low and high-level behavioral estimates were significantly predictive of the overall differentiation (*T* = 5.403, *p* = 0.001; and *T* = 7.928, *p* = 0.001). Even when the low-level ratings were regressed out of the differentiation measure, the high-level behavioral ratings still significantly predicted the remaining differentiation values (*T* = 3.318, *p* = 0.003). At the individual level, the behavioral ratings for high-level features correlated significantly with neurophysiological differentiation in 7 of the 9 participants, and in 3 of the 9 participants for low-level features.

We also examined whether measures of stimulus differentiation of the images correlated with neurophysiological differentiation. Stimulus differentiation was estimated by creating RDMs using three different distance measures: pixel to pixel correlation; mean luminosity; and Weibull statistics of spatial coherence and contrast energy (Scholte et al., 2009; Groen et al., 2012). The relationship between stimulus and neurophysiological differentiation was assessed using non-parametric permutation statistics as above. Pixel correlation was found not to be a significant predictor of differentiation (*T* = -1.485, *p* = 0.067). Stimulus overall luminosity differentiation was related to differentiation (*T* = 5.130, *p* = 0.001). Also, the Weibull distances within image categories also significantly predicted subsequent differentiation (*T* = 5.856, *p* = 0.001). Crucially, however, when this relationship was regressed out of the differentiation values, the meaningful versus meaningless distinction remained a significant predictor (*T* = 3.539, *p* = 0.002). In other words, even when stimulus differentiation is allowed to capture the initial variance, the basic phenomenal split accounts for a significant portion of the remaining variance. The same was found for the additional effect of the behavioral measures over and above the stimulus differentiation (*T* = 3.918, *p* = 0.001). At the individual level only 2 of 9 participants showed significant correlations to any of the stimulus differentiation measures.

### Novelty and Surprise

We calculated the differentiation with respect to predictability and novelty to examine whether the results found between the meaningful and meaningless images could be accounted for by these control factors. Two distinct categories were available for the analysis of short-term novelty: either the item was habituated to prior to the experiment start, or the image was a novel one. We could therefore measure the CDI based on this categorical distinction. For the case of predictability and familiarity there were three distinct conditions, so a single CDI is not possible. However, since the conditions can be ordered from low-to-high predictability or familiarity, we tested for monotonically increasing values using the spearman correlation coefficient. That is, we test for whether the intermediate levels of predictability or familiarity also have intermediate levels of differentiation. As before, these measures were also taken for 5000 permuted datasets to examine individual significance while one-sample *t*-tests over these values tested for group significance. At the group level, neither the habituation vs. novel (mean CDI = 0.007, *SE* = 0.008; *t*_8_ = 0.8191, *p* = 0.436) or levels of familiarity (mean CDI = -0.006, *SE* = 0.018; *t* = -0.3266, *p* = 0.752) were found to have significant effects on levels of differentiation. At the individual level, two participants showed significant CDI for short-term novelty, however, in different directions.

**FIGURE 7:**
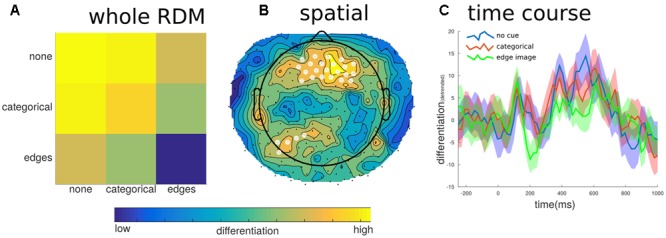
Differentiation to predictability levels. To investigate potential effects of image predictability we examined differentiation to images that were preceded by no cue, the categorical word cue, or the edge detected image. **(A)** Generally, images in which the content was highly predictable (edges images), showed the lowest differentiation when all channels and samples defined the state. **(B)** This effect was localized to fronto-central electrodes (significant electrodes shown with a white overlay); this region did not overlap with the posterior region of interest shown in **Figure 5A**. **(C)** The temporal breakdown of differentiation revealed no time ranges where there was a significant difference between the predictability levels of the target image.

For the different levels of predictability, we found a significant effect on differentiation at the group level (mean correlation = -0.099, *SE* = 0.041, *t* = -2.435, *p* = 0.041). The negative value indicates that when the images were the least predictable the neurophysiological differentiation was highest; whereas when the image was most predictable, following the prior exposure to the edge detected version of the image, the neurophysiological differentiation was significantly lower. At the individual level, only two participants showed significant effects, and two more showed trend levels. However, all but one participant had negative overall correlation values. In order to determine if this effect of predictability overlapped with the effects of meaningfulness we further broke down the EEG into its spatial and temporal parts and re-calculated the differentiation values. As **Figure 7** shows, there were some peak time points of the effect, particularly around 200 ms and again around 550 ms, however, none of these time points were found to be significantly different (e.g., mean CDI at 210 ms = -0.058, *SE* = 0.016; *t* = -3.545, *p* = 0.287). On the other hand, the spatial breakdown showed two clusters of channels with significant effects. The largest cluster consisted of 30 channels of the fronto-central electrodes (peak channel E20: *t* = -5.601, *p* < 0.001), while the second smaller cluster consisted of just five channels over the left-parietal area (peak channel E107: *t* = -7.230, *p* = 0.012). Therefore, there was no specific effects of differentiation for either factors involving stimulus novelty or familiarity. Moreover, the significant effects of differentiation with respect to predictability show different spatial and temporal properties compared to those for meaningful versus meaningless stimulus sets.

### Event-Related Potentials

**FIGURE 8:**
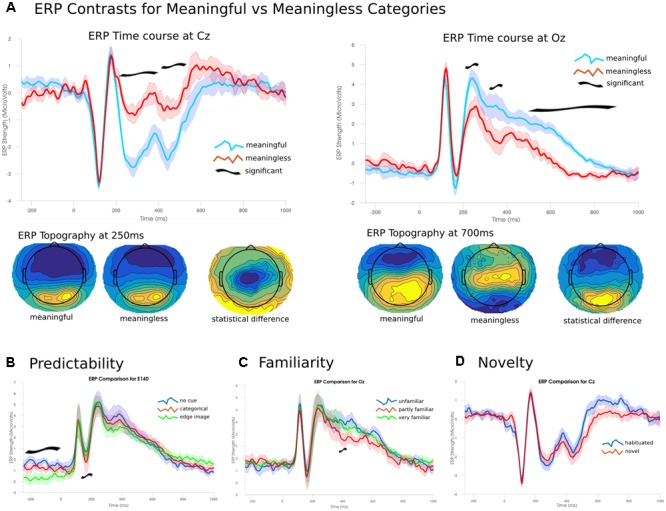
Event-related potentials and topographies. **(A)** The ERP time courses from -250 to 1000 ms at two electrode sites Cz and Oz (central and occipital, respectively). Significant differences emerged from 200 ms onward. Early differences were due to larger amplitudes for meaningful images compared to meaningless. Later differences were due to a shift in topographies between the two conditions. **(B)** Predictability of the target image lead to significant effects during the baseline period and around 200 ms. **(C)** Only a narrow time range around 390 ms showed a significant effect of familiarity; images rated as partly familiar showed lower posterior amplitudes. **(D)** No significant differences were found between habituated and novel images.

To establish the novelty of the DA analysis, it is important to contrast the observed changes in differentiation with changes in the ERP. ERP differences between images from the meaningful and meaningless stimulus sets showed essentially two large clusters of significant channels (*p* < 0.05), one posterior and the other fronto-central spanning much of the time course from 202 to 870 ms. The posterior cluster peaked at 710 ms at channel E137 (central-posterior; *t* = 12.511, *p* < 0.001), and corresponded to higher positive amplitudes for meaningful images. The fronto-central cluster peaked at 246 ms at channel E45 (just left of Cz; *t* = -12.101, p = 0.005), and corresponded to larger negative amplitudes for meaningful items. These differences arose from two fairly consistent topographies which showed an abrupt change at 530 ms (see **Figure 8**).

A three-way ANOVA (corrected using TFCE), examining the effect of the predictability of each image found two time ranges of significant differences. The largest difference in the ERPs was found during the baseline period, prior to the presentation of the image between 250 and 26 ms in right posterior channels (peak channel E140; *f* = 31.314, *p* = 0.002) where the highly predictive cues showed a stronger negative amplitude than either of the other two conditions. The other significant cluster was found between 160 and 240 ms for central-posterior channels (peak channel E136 at 194 ms; *f* = 38.642, *p* = 0.009). Here, stronger negative amplitudes were found for images preceded by the categorical cue, with intermediate amplitudes for no cue and the lowest amplitudes for the most predicable images. The ANOVA examining only the images rated for their familiarity showed only a single significant channel over two samples at 390–394 ms (*f* = 20.224, *p* = 0.048). Here, familiar images showed a significantly lower amplitude compared to the either unfamiliar or only partly familiar items which showed no differences. Analysis of the effect for short-term novelty found no significant differences between images that were seen immediately prior to the experiment and those that were novel to the participant.

## Discussion

The aim of this study was to develop an analysis technique to quantify the meaningfulness of sets of stimuli through the analysis of neurophysiological differentiation – the distance between neural states triggered by different stimuli. We assume that a stimulus set is meaningful for a particular subject if different stimuli within the set elicit different phenomenal concepts, typically high-level ones (phenomenal differentiation), and that neurophysiological differentiation is a prerequisite for phenomenal differentiation. We used HD-EEG to analyze responses to several categories of meaningful natural images and artificially generated meaningless images. The results show that neurophysiological differentiation was higher for the meaningful set of images than for a less meaningful set of images both at the group level and for individual subjects. We also analyzed the characteristic spatio-temporal profile of neurophysiological differentiation as a marker of meaningfulness, and examined the role of potential confounding factors such as novelty, familiarity, and predictability.

### Meaningfulness, Phenomenal Differentiation, and Stimulus Differentiation

A key premise of this work is that phenomenal differentiation can be high or low, for the same level of stimulus differentiation, depending on the meaningfulness of different stimuli to the subject. The phase-scrambled images employed in the present study are a case in point. While at the stimulus level each phase-scrambled image is just as different from the others as is each natural image, for each subject phenomenal differentiation was high for natural images and low for phase-scrambled images, as indicated by behavioral ratings. Crucially, neurophysiological differentiation as measured by HD-EEG responses mirrored phenomenal differentiation. This is supported by recent evidence from MEG showing that perceptual similarity judgments predicted neural activity better than stimulus properties as early as 80 ms after image presentation (Wardle et al., 2016). Or that combined models of image statistics and semantic models can account for the neurophysiological response better than either alone (Clarke et al., 2015). Our results support these notions in that even the simple and trivial distinction of meaningful versus meaningless image categories is a significant predictor of neurophysiological differentiation beyond what the differentiation of the stimulus properties can account for.

A dissociation between stimulus differentiation and phenomenal differentiation is also suggested by certain perceptual illusions. For example, visual metamers and change blindness illustrate how substantial changes in the physical stimuli may not be perceived (Busch et al., 2010; Freeman and Simoncelli, 2011). Conversely, in the checker shadow illusion, two identical shades of gray are perceived as different due to their background (Adelson, 1995). Thus, DA provides a tool to assess subjective meaningfulness by measuring the neurophysiological differentiation that supports phenomenal experience. Thus, one can say that the relationship between the physical stimulus properties and neurophysiological differentiation is determined by whether the stimulus leads to a different subjective experience.

### Spatio-Temporal Breakdown

Spatial breakdown of CDI showed that electrodes in the central-posterior region were more likely to show higher differentiation to meaningful images. This localization is consistent with the visual nature of the stimuli used in this study, suggesting that the different images within meaningful categories evoke distinct activation patterns in high-level visual cortical areas, while different images within less meaningful categories tend to evoke similar activation patterns. No channel displayed a negative CDI, indicating that the pattern of underlying neural activity was always more diverse (significantly or not) for meaningful images, no matter which region was examined. The topography of visual differentiation obtained here is similar to that highlighted by recent work using multivariate decoding for object recognition and categorization (Simanova et al., 2010; Kaunitz et al., 2011; Kaneshiro et al., 2015). Of note, Kaunitz et al. (2011) found that decoding was only able to discriminate between image sets when they had been perceived (in a flash suppression paradigm).

Temporal breakdown found two peaks in differentiation and CDI: an early peak (110 ms) and a late one (300+ ms). A possible interpretation of the two peaks is provided by the distinction between phenomenal and access consciousness (Block, 2007; Lamme, 2010). The high values of differentiation and CDI at 110 ms would be compatible with the idea that different phenomenal contents are experienced for different meaningful images during this time, in line with other studies that show content-related activations in posterior cortex at similar latencies (Pitts et al., 2014; Tapia et al., 2014). The differentiation peak after 280 ms could be related to the subsequent access and reflection about the experienced content through attentional mechanisms (Lamme, 2003). Thus, prefrontal regions may reactivate the posterior region thereby amplifying the different activity patterns supporting the distinct phenomenal experiences (Ro et al., 2003; Chambers et al., 2013). Reflection on particular features of each experience would then be likely to evoke higher-level, abstract concepts supported by additional cortical regions.

### Relationship to Event-Related Potentials

A dissociation is demonstrated between of DA and ERP analysis, and these differences start at the conceptual level. In ERP analysis, many trials are averaged together to identify the time-dependent amplitude of an underlying evoked potential, and deviation from this waveform is seen as ‘noise’ within the trial. On the other hand, DA is specifically interested in the differences between trials without reference to the common underlying signal. In our approach, these differences are not seen as noise, but rather unique neurophysiological states that support differences in subjective experience.

A conventional amplitude analysis of the ERP associated with meaningful and meaningless images highlighted two time intervals that were significantly different. The amplitude difference at 250–500 ms over central channels may be related to the visual-awareness-negativity (VAN) (Pitts et al., 2014; Shafto and Pitts, 2015). The VAN is obtained by contrasting the conscious perception of an image with an unperceived, masked variant of it (Railo et al., 2011). This contrast resembles aspects of the meaningful and meaningless images used in the present study. In these types of experiments, one could say the target stimuli have generally higher phenomenal differentiation (e.g., faces), while the unperceived masks have low phenomenal differentiation. Thus, the ERP differences we find here (i.e., VAN) are consistent with such a reframing of the classical perception paradigms. In the future, applying DA to perception tasks may provide more direct evidence for this observation.

The VAN has been related to possible top-down effects of object-based attention (Shafto and Pitts, 2015), which may indicate later attentional access amplifying earlier phenomenal distinctions. This interpretation would fit with the observation of a second differentiation peak (after 280 ms), suggesting that, if images are phenomenally perceived as different, they may trigger top-down attentional facilitation that enhances the overall response and subsequently amplifies their differentiation. By contrast, the lack of ERP changes at the time window of the first peak in differentiation (at 110 ms) is consistent with the idea differences in pattern of activity are not necessarily accompanied by differences in amplitude, yet they may underlie differences in phenomenal perception. Indeed, previous research has shown that even for basic contrasts between face and house stimuli, level of neural activity is insufficient to classify the stimuli, and that the patterns of activity are crucial (Hanson and Halchenko, 2007) but see (Roeber et al., 2008; Clarke et al., 2013).

Later ERP differences (500–800 ms) show different topographies for each condition and thus likely represent distinct neural sources, possibly related to the late-positive-potential (LPP; Koivisto et al., 2009). The LPP has been associated with post-perceptual activity, such as task-related activity or reportability of target stimuli (Railo et al., 2011; Dehaene, 2014). However, the observation that CDI values are also high during this later time range suggests that these late responses may also correspond to phenomenal differentiation, or at least to highly differentiated post-perceptual processes.

### Differentiation vs. Novelty, Familiarity, and Predictability

An explicit goal of this study was to investigate whether the results of DA would be confounded by other factors, such as novelty, familiarity, and predictability of stimuli. We found no effect for the short-term novelty of stimuli, as response differentiation was not affected by whether the image had already been presented or not. The long-term familiarity of stimuli, as assessed by subjective ratings, also had no effect on response differentiation, although it did affect the amplitude of late ERP responses. While our paradigm does not rule out the possibility that other forms of novelty and familiarity, for example the sequential presentation of the same image, or the presentation of highly personal images (Charest et al., 2014) could have some effect, they clearly indicate that measuring response differentiation reflects primarily the extent to which meaningfully different stimuli trigger different neural activity patterns underlying phenomenal differentiation.

Highly predictable images (edge version of the image), showed reduced neurophysiological differentiation; irrespective of image category. However, spatially the effect of predictability was strongest in fronto-central regions and did not overlap with the centro-posterior regions involved in differentiation to meaningful vs. meaningless images. Moreover, there was no specific time range for the effects of image predictability on differentiation, suggesting a weaker and dispersed influence. With respect to ERP, predictability had an impact at around 200 ms (**Figure 7**), suggesting a link to the VAN and object-based attention (Horstmann, 2015).

Our current experimental setup cannot rule out some potential confound of the imbalance in the stimulus types, with the majority of images being of the generally meaningful sort (7 categories compared to 3 meaningless). This may have led to a certain general unexpectancy of seeing a meaningless image as the experiment went on. However, given the explicit findings of short-term predictability described above, our data suggests that the effect would be higher differentiation, rather than lower, for the meaningless categories. This imbalance could nevertheless have led to a bias in attention for the more abundant meaningful images. The potential modulating effect of attention on neurophysiological differentiation, whether explicit or implicit, is of great interest and should be explored in future work.

### Potential Impact and Future Work

The present findings using HD-EEG, together with previous results with fMRI (Boly et al., 2015), establish the feasibility of DA as a tool to investigate neurophysiological differentiation as an index of phenomenal differentiation and thereby of the meaningfulness of set of stimuli for an individual subject. Importantly, DA can provide an objective measure of meaningfulness without requiring *a priori* knowledge about which aspects and categories of a stimulus set may be meaningful to a subject, without the need for *a priori* specification of regions or time points of interest, and without depending on behavioral reports. These features of DA makes it potentially relevant for investigating stimulus meaningfulness in subjects with cognitive skills that deviate from the norm and in non-human species. DA might also be useful as a neurophysiological correlate of learning, to the extent that learning implies an increasing ability to differentiate a set of previously undifferentiated stimuli through the development of new concepts and associated activity patterns. DA may also prove useful in investigating the presence of consciousness in unresponsive subjects, such as patients in a vegetative state (unresponsive wakefulness syndrome; Gosseries et al., 2014). In such patients, the finding of substantial levels of neurophysiological differentiation in response to meaningful but not meaningless sets of stimulus would strongly suggest the presence of accompanying phenomenal differentiation and therefore of consciousness (Marshall et al., 2016). In this respect, DA can be distinguished from measures of stimulus-induced complexity (e.g., Casali et al., 2013; Gosseries et al., 2015). While these responses may be complex in their spatio-temporal evolution, they can nonetheless show low differentiation if different stimuli produce similar responses. DA may also be helpful in identifying the neural substrate of consciousness in healthy subjects, under the assumption that spatio-temporal regions of high neurophysiological differentiation for meaningful stimuli will also mark the boundaries of the cortical regions that support their phenomenal perception. In this respect, a wide, multi-modal stimulus set, such as movies, would be well-suited for examining the boundaries of the neural structures supporting conscious experience as a whole.

## Conclusion

Differentiation of EEG activity, as measured by the within-category Euclidean distance, provides a novel way to measure to what extent a particular stimulus set elicits distinct patterns of neural activity, which we argue reflect its overall meaningfulness to a particular subject. Here we show that neurophysiological differentiation is high for meaningful images and low for meaningless ones, both at the group level and at the level of individual subjects, and that neurophysiological differentiation correlates with each subject’s behavioral rating of meaningful differences among the images. Crucially, these results are not better explained by the stimulus properties of the image set, the novelty or familiarity of the images, or the degree to which the images are predictable. Moreover, changes in differentiation doubly dissociate with respect to ERP changes. Future work should examine neurophysiological differentiation with stimuli that go beyond the visual modality as well as with streamed, naturalistic inputs, such as movies.

## Author Contributions

AM: Design of the work; acquisition of data; analysis; interpretation of data; and manuscript preparation. WM: Analysis; interpretation of data; and manuscript review. GT: Design of the work; interpretation of data; and manuscript preparation.

## Conflict of Interest Statement

The authors declare that the research was conducted in the absence of any commercial or financial relationships that could be construed as a potential conflict of interest.
