# Supplementary figures and images for: EEG Differentiation Analysis and Stimulus Set Meaningfulness
Source: Front Psychol. 2017 Oct 6;8:1748. doi: 10.3389/fpsyg.2017.01748 (PMC5635725; doi:10.3389/fpsyg.2017.01748)

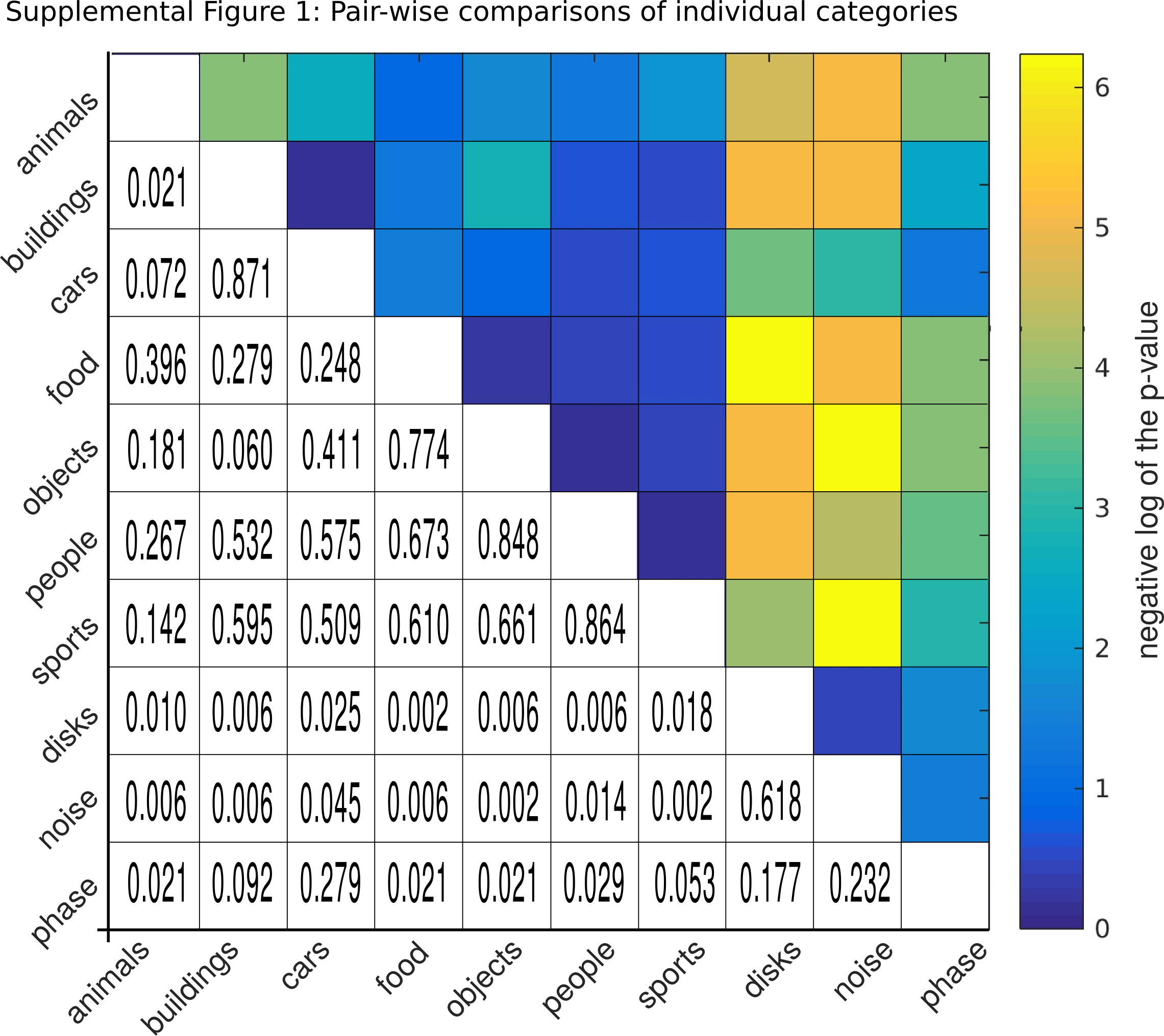

Supplement: Supplementary file 1 [file Image_1.TIF]
